# Comparison of ROX, HROX, and delta-HR indices to predict successful weaning from high-flow oxygen therapy in hospitalized patients with COVID-19 pneumonia

**DOI:** 10.1371/journal.pone.0297624

**Published:** 2024-02-15

**Authors:** Pitchayapa Ruchiwit, Jaturong Madua, Narongkorn Saiphoklang

**Affiliations:** Department of Internal Medicine, Division of Pulmonary and Critical Care Medicine, Thammasat University, Klongluang, Pathum Thani, Thailand; University of Palermo, ITALY

## Abstract

**Background:**

High-flow nasal cannula (HFNC) therapy is commonly used to treat acute respiratory failure in patients with COVID-19 pneumonia. However, predictors of successful weaning from HFNC in these patients has not been investigated.

**Objective:**

To assess predictors of successful separation from HFNC in patients with COVID-19 pneumonia.

**Methods:**

We conducted a retrospective cohort study at a university hospital in Thailand. Patients with COVID-19 pneumonia requiring HFNC therapy between April 2020 and June 2022 were included. ROX index was defined as the ratio of oxygen saturation (SpO_2_) / fraction of inspired oxygen (FiO_2_) to respiratory rate. Heart-ROX (HROX) index was defined as ROX multiplied by heart rate (HR) improvement. HR improvement (delta-HR) was defined as a percentage of the difference between the baseline HR and the morning HR at HFNC weaning day 1 divided by the baseline HR. Weaning success was defined as ability to sustain spontaneous breathing after separation from HFNC without any invasive or non-invasive ventilatory support for ≥48 hours or death.

**Results:**

A total of 164 patients (54% male) were included. Mean age was 61.1±16.1 years. Baseline SpO_2_/FiO_2_ was 265.3±110.8. HFNC weaning success was 77.4%. The best cut-off value of ROX index to predict HFNC weaning success was 7.88 with 100% sensitivity, 97.3% specificity, and area under the ROC curve (AUC) of 0.98 (95% CI: 0.937–1.000, p<0.001). The best cut-off value of delta-HR 3.7 with 88.2% sensitivity, 75.7% specificity, and AUC of 0.83 (95% CI: 0.748–0.919, p<0.001). The best cut-off value of HROX index was 59.2 with 88.2% sensitivity, 81.1% specificity, and AUC of 0.89, (95% CI: 0.835–0.953, p<0.001).

**Conclusions:**

The ROX index has the highest accuracy for predicting successful weaning off HFNC treatment in patients with COVID-19 pneumonia. While HROX and delta-HR indices can serve as alternative tools, it is recommended to verify these indices and determine the optimal cut-off value for determining separation from HFNC therapy through a large prospective cohort study.

**Trial registration:**

**Clinicaltrials.in.th number:** TCTR20221108004.

## Introduction

Coronavirus Disease 2019 (COVID-19) is an infectious disease that is still spreading. Most patients have respiratory infection symptoms such as a cough, runny nose, sore throat, and fever. Some people have COVID-19 pneumonia, resulting in decreased oxygen levels in the blood. These patients need oxygen supply via an oxygen cannula, a high-flow nasal cannula (HFNC), or non-invasive ventilation, depending on oxygen concentration and the severity of pneumonia [1, 2]. Although the use of non-invasive respiratory support in acute respiratory failure due to viral infection and pandemics is still debated, without evidence in favor or against their use at the early stage of the pandemic, non-invasive respiratory support strategies were widely and variably used during the pandemic [3].

If the inflammatory process continues, respiratory failure can occur, requiring invasive mechanical ventilation and increasing the risk of death [4]. HFNC has been widely used during the COVID-19 pandemic with different reported outcomes [5]. A recent systematic review and meta-analysis [4] indicated that HFNC may reduce the intubation rate compared to conventional oxygen therapy. Nevertheless, there is still conflicting evidence regarding the benefits of its use [6, 7]. However, delayed intubation can have adverse effects on patients [8]. Therefore, proper intubation and use of HFNC can make treatment more effective. The ROX index, defined as the ratio of SpO_2_/FiO_2_ to respiratory rate, is used to predict successful HFNC treatment [9]. Treatment success is determined by a ROX index value of 12.7 with 85% sensitivity and 41% specificity [10]. Humidified high-flow nasal cannula (HFNC) has been identified as a safe and effective therapy for hypoxemic acute respiratory failure in COVID-19 patients [11]. It reduces respiratory rate and increases oxygen saturation, potentially decreasing the need for mechanical ventilation. However, delaying intubation through non-invasive approaches may lead to worse outcomes [8].

Despite the clinical benefits of HFNC, previous studies have not fully explored predictors of weaning success in COVID-19 pneumonia patients. Previous research has found the ROX index to be a useful indicator of disease severity and predictor for intubation. A value of less than 4.88 is associated with intubation, with an HR of 0.273, CI95% 0.121–0.618, and p value of .002 [9].

This study aims to identify new predictors of successful weaning from HFNC among COVID-19 patients hospitalized with pneumonia. We will focus on two novel predictors: 1) the HROX index, which is calculated by using the ROX index in conjunction with heart rate; 2) the HR improvement (delta-HR) index, which is defined as the percentage difference between the baseline heart rate and the morning heart rate on the first day of HFNC weaning, divided by the baseline heart rate.

## Methods

### Study design and participants

This retrospective cohort study was conducted at Thammasat University Hospital in Thailand. Patient data between 1^st^ April 2020 and 30^th^ June 2022 were extracted through electronic medical records, nursing record, and ICD-10. The data were accessed for research purposes in July 2022. The inclusion criteria were patients aged 18 years or older, hospitalized in respiratory wards, including both ICU and non-ICU wards with COVID-19 pneumonia, requiring HFNC treatment within 24 hours of admission, and with confirmed diagnosis by a positive SARS-CoV-2 reverse transcription-polymerase chain reaction test and infiltrates observed on chest radiographs. Patients receiving HFNC treatment after extubation, those with cardiac arrhythmias, those taking medications that affect heart rate (such as beta-blockers, non-dihydropyridine calcium channel blockers, amiodarone, or digoxin), and those with advanced-stage disease or a “do not intubate order (DNI)” were excluded.

Clinical data including demographics, co-morbidities, clinical characteristics, laboratory results, baseline oxygenation level and SpO_2_/FiO_2_ ratio, respiratory rate, baseline heart rate before HFNC weaning, morning heart rate on the first day of HFNC weaning, and the success or failure of weaning from HFNC, were analyzed.

Ethic approval was obtained from the Human Research Ethics Committee of Thammasat University (Medicine), Thailand (IRB No. MTU-EC-IM-0-065/65, COA No. 150/2022), in full compliance with international guidelines such as Declaration of Helsinki, The Belmont Report, CIOMS Guidelines and the International Conference on Harmonisation-Good Clinical Practice (ICH-GCP) (see S1 File). All methods were performed in accordance with these guidelines and regulations. This study was registered in the Thai Clinical Trials Registry (TCTR); thaiclinicaltrials.org (number: TCTR20221108004). Written informed consent was waived because this study was a retrospective study.

### Procedures and outcomes

The primary outcomes of this study were to determine the best cut-off values of the ROX, HROX, and delta-HR indexes for predicting weaning success in patients with COVID-19 pneumonia who received HFNC treatment. The ROX index was calculated as the ratio of SpO_2_/FiO_2_ to respiratory rate at 1 hour before HFNC weaning, while the HROX index was defined as ROX multiplied by heart rate (HR) improvement. HR improvement (delta-HR) was calculated as the percentage difference between the baseline HR and the morning HR on the first day of HFNC weaning, divided by the baseline HR. Weaning success was defined as the ability to sustain spontaneous breathing after separation from HFNC without any invasive or non-invasive ventilatory support for at least 48 hours, or until death.

### Statistical analysis

Data were expressed as number (%) and mean ± standard deviation. The chi-squared test was used to compare categorical data between the successful weaning group and the failed weaning group. The student’s t-test was used to compare continuous data between the two groups. A two-sided p-value < 0.05 was considered statistically significant. The ROC curve with sensitivity/specificity for each cut-off value was drawn, and the area under that curve was calculated. Statistical analyses were performed using SPSS version 26.0 software (IBM Corp., Armonk, NY, USA).

## Results

**Fig 1:**
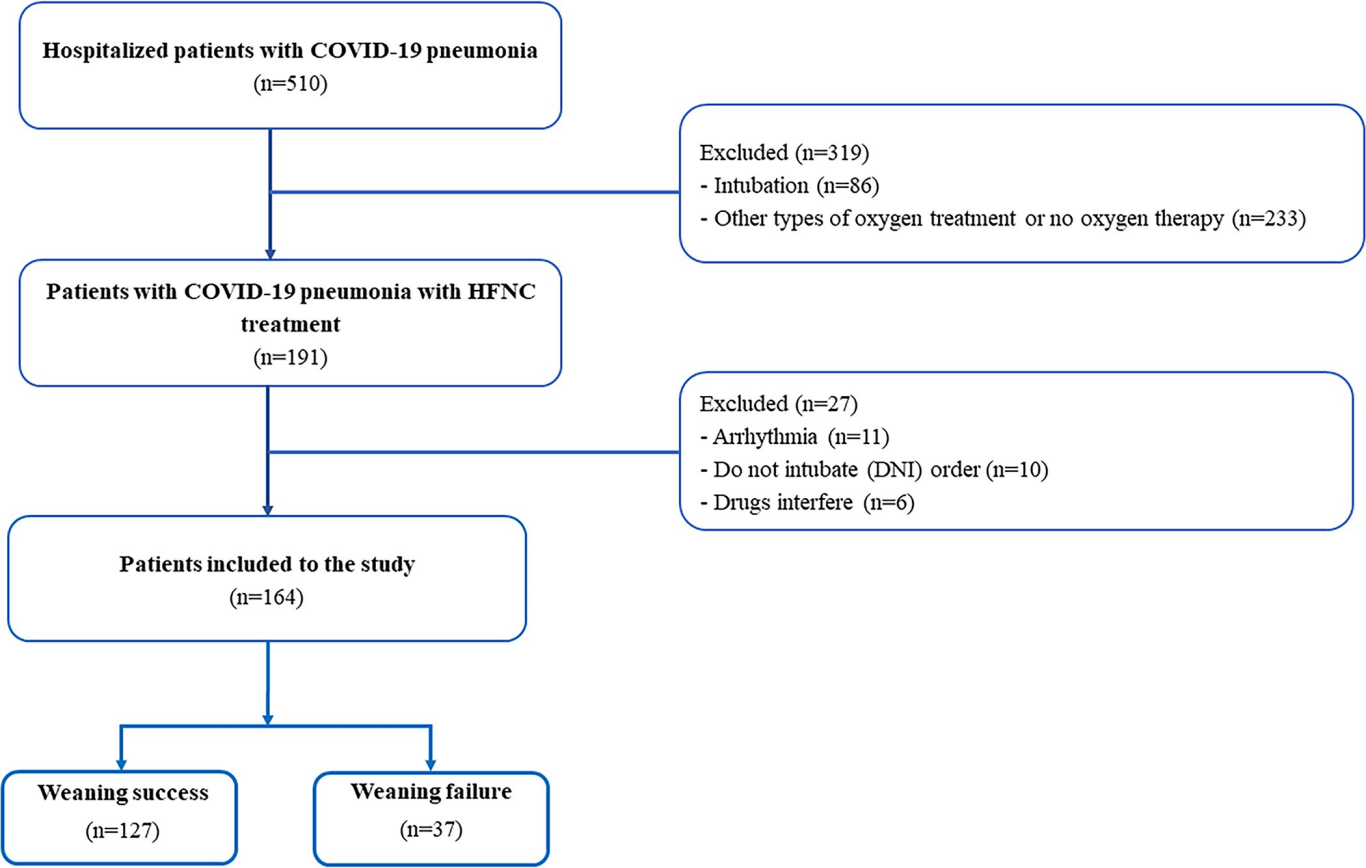
Flowchart of recruitment to the study for hospitalized patients with COVID-19 pneumonia.

Out of the 510 hospitalized patients with COVID-19 who were screened, 191 had pneumonia that required HFNC treatment. Of these, 27 patients included a “do not intubate order (DNI)” were excluded (see Fig 1). The final analysis included a total of 164 patients. Fifty-four percent of the patients (n = 89) were male, and the mean age was 61.1±16.1 years. The baseline SpO_2_/FiO_2_ was 265.3±110.1. The most common comorbidities were hypertension (present in 47.6% of patients), diabetes (36%), and dyslipidemia (33.5%) (see Table 1). Of these, 37 patients (22.6%) failed to wean off HFNC, and 10 patients in this group died.

**Table 1:** Baseline characteristics of hospitalized patients with COVID-19 pneumonia receiving high flow nasal cannula treatment. Data shown as n (%) or mean±SD.

Characteristic	Overall	Weaning success	Weaning failure
	(n = 164)	(n = 127)	(n = 37)
Age, years	61.13 ± 16.1	59.44 ± 16.6	66.92 ± 12.8
Male	89 (54.3)	73 (57.5)	16 (43.2)
BMI, kg/m^2^	27.28 ± 5.9	30.27 ± 32.4	27.33 ± 4.7
Respiratory rate (breaths/minute)	21.2 ± 5.2	19.3 ± 3.3	27.8 ± 5.4
FiO_2_	0.37 ± 0.11	0.33 ± 0.07	0.52 ± 0.09
SpO_2_	96.15 ± 4.0	97.65 ± 2.0	95.0 ± 1.4
**Scores**			
SOFA score	1.8 ± 1.0	1.76 ± 1.1	2.24 ± 0.8
APACHE II score	6.0 ± 3.6	5.46 ± 3.5	8.0 ± 3.5
SpO_2_/FiO_2_	265.3 ± 110.1	310.6 ± 78.3	189.6 ± 33.9
ROX index	13.87 ± 7.42	16.70 ± 5.79	7.16 ± 2.53
**Labs**			
Creatinine, mg/dL	1.3 ± 1.9	1.3 ± 1.9	1.4 ± 1.9
Leucocyte count, 10^3^/μL	7.7 ± 4.2	7.6 ± 3.8	8.1 ± 5.6
Platelet count, 10^3^/ μL	229.2 ± 115.3	223.0 ± 109.7	212.9 ± 104.9
CRP, mg/L	82.6 ± 69.8	75.4 ± 70.2	86.0 ± 73.3
**Comorbidities**			
Hypertension	78 (47.6)	54 (42.5)	24 (64.9)
Diabetes	59 (36)	43 (33.8)	16 (43.2)
Dyslipidemia	55 (33.5)	42 (33.1)	13 (35.1)
Chronic kidney disease	14 (8.5)	10 (7.9)	4 (10.8)
Benign prostatic hyperplasia	6 (3.7)	4 (3.1)	2 (5.4)
Gout	6 (3.7)	4 (3.1)	2 (5.4)
COPD	4 (2.4)	4 (3.1)	0
Cerebrovascular disease	4 (2.4)	2 (1.6)	2 (5.4)
Asthma	3 (1.8)	3 (2.7)	0
Cardiovascular disease	2 (1.2)	1 (0.8)	1 (2.7)

Data shown as n (%) or mean ± SD

kg = kilogram, m = meters, COPD = chronic obstructive pulmonary disease, SpO_2_ = arterial oxygen saturation, APACHE II = Acute Physiology And Chronic Health Evaluation, SOFA = Sequential Organ Failure Assessment, FiO_2_ = fraction of inspired oxygen, ROX index = respiratory rate and oxygenation index, mg = milligram, dL = decilitre, μL = microlitre, CRP = C-reactive protein, L = litre

### ROX, HR improvement (Delta-HR), and HROX indices

**Fig 2:**
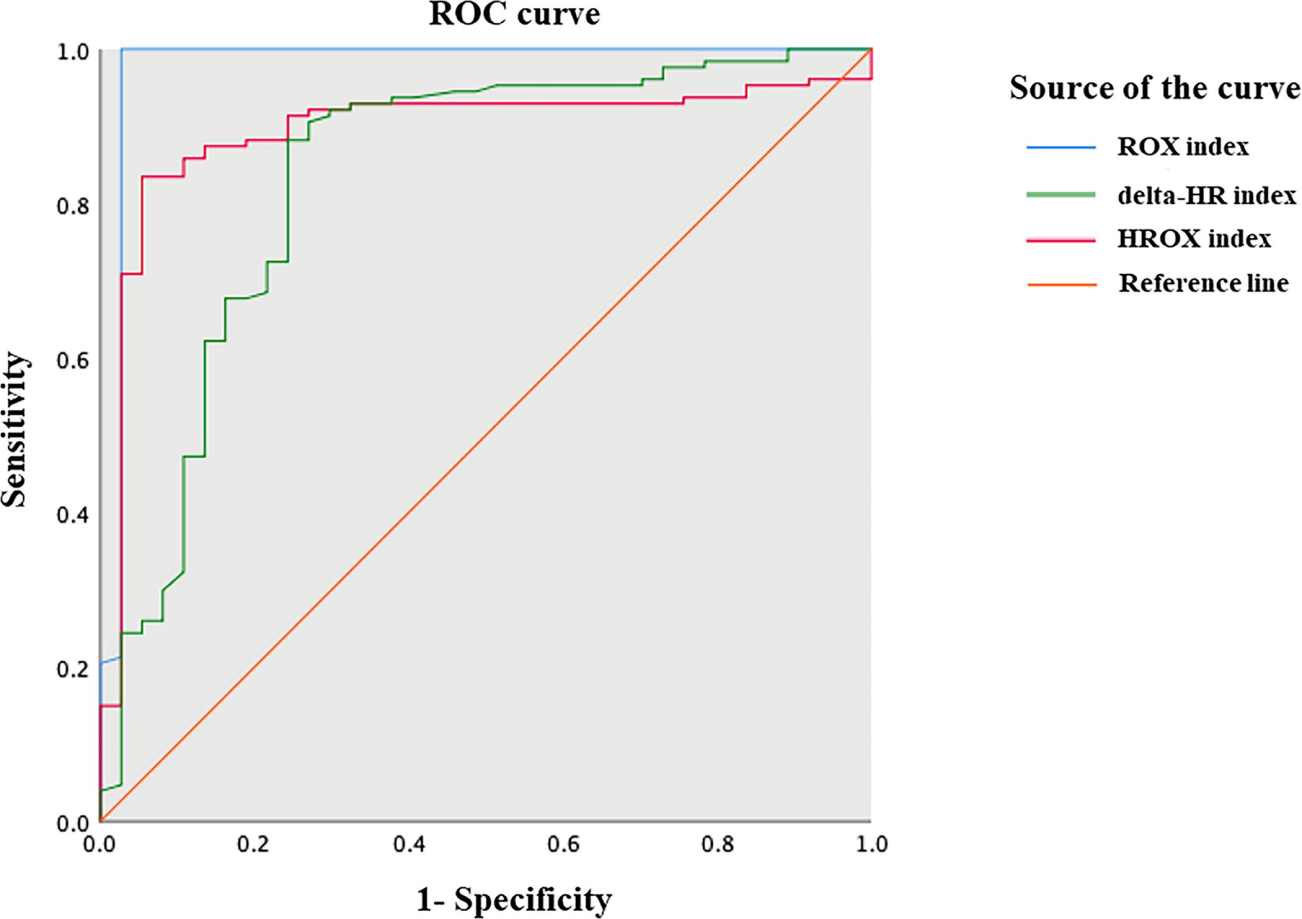
The receiver operating characteristic (ROC) plot of ROX, HR improvement (delta-HR), and HROX indices predicting successful weaning from high-flow nasal cannula therapy. The best cutoff values of ROX, delta-HR, and HROX indices are 7.88, 3.7 and 59.2, with the area under the ROC curve of 0.98, 0.83 and 0.89, respectively.

The proportion of COVID-19 pneumonia patients who successfully weaned off HFNC was 77.4%. The best cut-off value for the ROX index at 1 hour before HFNC weaning in predicting HFNC weaning success was 7.88, with a sensitivity of 100%, specificity of 97.3%, positive predictive values (PPV) of 99.2%, negative predictive values (NPV) of 100%, and an area under the receiver operating characteristic curve (AUC) of 0.98 (95% CI: 0.937–1.00, p<0.001). For the delta-HR index, the best cut-off value to predict HFNC weaning success was 3.7, with a sensitivity of 88.2%, specificity of 75.7%, PPV of 92.6%, NPV of 65.2%, and an AUC of 0.83 (95% CI: 0.748–0.919, p<0.001). Regarding the HROX index, the best cut-off value to predict HFNC weaning success was 59.2, with a sensitivity of 88.2%, specificity of 81.1%, PPV of 94.1%, NPV of 66.7%, and an AUC of 0.89 (95% CI: 0.835–0.953, p<0.001).(see Fig 2 and Table 2).

**Table 2:** The best cut-off values of ROX, HR improvement (delta-HR), and HROX indices predict HFNC weaning success.

Variable	Cut-off value	AUC	95% CI	Sensitivity (%)	Specificity (%)	PPV (%)	NPV (%)	P-value
ROX index	7.88	0.98	0.937–1.00	100	97.3	99.2	100	<0.001
Delta-HR index	3.7	0.83	0.748–0.919	88.2	75.7	92.6	65.2	<0.001
HROX index	59.2	0.89	0.835–0.953	88.2	81.1	94.1	66.7	<0.001

AUC = area under the ROC curve, CI = confidence interval, NPV = negative predictive values, PPV = positive predictive values

## Discussion

Our study is the first cohort investigation of predictors for successful weaning off HFNC treatment in hospitalized patients with COVID-19 pneumonia. The findings show that the ROX index had both the highest sensitivity, specificity and AUC, followed by the HROX and delta-HR indices.

The ROX index is a valuable tool in predicting intubation after HFNC treatment in patients with acute hypoxic respiratory failure. Numerous studies have demonstrated a significant correlation between ROX index and deteriorating outcomes. A multicenter, prospective observational cohort study by Roca O. et al. [12], conducted over two years, found that the ROX index was effective in identifying patients at high risk of intubation. Of the 191 patients treated with HFNC, 68 (35.6%) required intubation. The study also showed that the prediction accuracy of the ROX index increased over time (AUC: 0.679 at 2 h, 0.703 at 6 h, and 0.759 at 12 h). Additionally, an ROX greater than 4.88 measured at 2 h after HFNC initiation had a significant association with a lower risk for intubation [12]. In line with the findings of the retrospective cohort study involving 129 patients with COVID-19 pneumonia conducted by Patel M et al. [13], the study indicated that an ROX index value of less than 5 at the initiation of HFNC suggested a progression to invasive mechanical ventilation (odds ratio 2.137, P = .052). Furthermore, any subsequent decrease in the ROX index value after HFNC initiation was predictive of intubation (OR 14.67, P < .001). Another retrospective review examined the medical records of 105 patients with COVID-19 who were treated with HFNC and successfully withdrawn from HFNC, conducted by Hu Ming et al. [14]. The study found that the ROX index after 6 hours of HFNC initiation (AUROC, 0.798) demonstrated good predictive capacity for HFNC outcomes, with a ROX index greater than 5.55 at 6 hours after initiation significantly associated with HFNC success (OR, 17.821; 95% CI, 3.741–84.903, p<0.001). A multicenter, prospective study by Mellado-Artigas R et al. [15] demonstrated factors associated with endotracheal intubation in patients with COVID-19 pneumonia. The study revealed that the ROX index, SOFA score, and pH were predictors of endotracheal intubation. The SOFA score and ROX index showed excellent performance with an AUC of 0.88 (95% CI 0.80–0.96). Another retrospective observational study by Maeva Rodriguez et al. [10] conducted over two years found that FiO_2_ ≤ 40% and ROX index ≥ 9.2 were predictors of successful separation from HFNC in patients with pneumonia and acute respiratory failure.

In a prospective observational cohort study by Goh KJ et al. [16], 145 patients with acute hypoxemic respiratory failure treated with HFNC were followed a planned extubation. The study showed that lower ROX and ROX-HR index values recorded at different time points within 48 hours were associated with HFNC failure. Between the first 12 hours, the highest AUC for both indices occurred at 10 hours, with 0.723 and 0.739 for the ROX and ROX-HR indices, respectively. At 10 hours, a ROX-HR index above 6.80 was associated with a lower risk of HFNC failure (hazard ratio 0.301).

Our study is the first to evaluate the effectiveness of combining heart rate with ROX (HROX) or subtracting the baseline heart rate from the heart rate during HFNC treatment (delta-HR) in predicting HFNC weaning outcomes in patients who have recovered from COVID-19 pneumonia. We found that the HROX index had significant predictive value in HFNC weaning success with the best cut-off value of 59.2. Similarly, the delta-HR index showed predictive value with the best cut-off value of 3.7. However, the ROX index had the highest sensitivity and specificity in predicting HFNC weaning outcomes with the best cut-off value of 7.88.

We hypothesized that incorporating heart rate into the ROX formula would improve accuracy and predict successful weaning from HFNC. Given the challenges of assessing patients during the COVID-19 outbreak, particularly through physical examinations, the decision to wean HFNC is largely dependent on vital signs and symptoms. A notable strength of these indexes is that they are rapid bedside tools that are non-invasive, fast, easily reproducible, inexpensive, and highly accessible. Therefore, they can be used in low-resource settings and might be calculated by nurses, which is crucial during the pandemic. Our findings suggest that the HROX and delta-HR indices could serve as effective tools for the early prediction of successful weaning from HFNC therapy among hospitalized patients with COVID-19 pneumonia. However, the ROX index demonstrated the highest accuracy, sensitivity, and specificity compared to the HROX and delta-HR indices. Adding heart rate assessment does not add value to the ROX index, as heart rate is a variable that can change based on various conditions such as activity, emotions, illness, etc.

The study had a few limitations. Firstly, the timing of the heart rate record to calculate HROX or delta-HR indices may have affected their accuracy. As heart rate is a sensitive factor, patients with arrhythmias or those taking heart rate control medications (e.g., beta blockers) on the day of HFNC weaning may not have had accurate indices to predict the outcome of HFNC weaning. The timing of the heart rate record is debated, and we chose 2 a.m. as patients have less activity during that time, leading to less interference for heart rate analysis. Secondly, the parameters involved in the ROX index can vary throughout the day or in different clinical situations, which may lead to possible biases. Recording parameters at the same time every day when patients had no activity for treatment decreased interference with result analysis. Thirdly, for the calculation of HR improvement and HROX, patients with arrhythmia or those taking medications such as beta-blockers were excluded. This exclusion may limit the generalizability of the results, as hospitalized patients often have chronic cardiovascular diseases. These results should be confirmed in future validation cohorts before proposing the use of these indices for predicting successful weaning from HFNC in patients with COVID-19 pneumonia. However, our results suggest that monitoring COVID-19 pneumonia patients weaning from HFNC therapy with the ROX, HROX, and delta-HR indices could predict clinical outcomes.

## Conclusions

The ROX index has the highest accuracy for predicting successful weaning off HFNC treatment in patients with COVID-19 pneumonia. While HROX and delta-HR indices can serve as alternative tools, it is recommended to verify these indices and determine the optimal cut-off value for determining separation from HFNC therapy through a large prospective cohort study.

## Supporting information

S1 File Dataset of patients with COVID-19 pneumonia. (XLSX)
